# Brainstem phenotype of cathepsin A–related arteriopathy with strokes and leukoencephalopathy

**DOI:** 10.1212/NXG.0000000000000165

**Published:** 2017-07-06

**Authors:** Yun Tae Hwang, Rahul Lakshmanan, Indran Davagnanam, Andrew G.B. Thompson, David S. Lynch, Henry Houlden, Nin Bajaj, Sofia H. Eriksson, Doris-Eva Bamiou, Jason D. Warren

**Affiliations:** From the Dementia Research Centre (Y.T.H., J.D.W.), Department of Neurodegenerative Disease (A.G.B.T.), Department of Molecular Neuroscience (D.S.L., H.H.), UCL Institute of Neurology, and UCL Ear Institute (D.-E.B.), University College London; Lysholm Department of Neuroradiology (R.L., I.D.) and Department of Clinical and Experimental Epilepsy (S.H.E.), National Hospital for Neurology and Neurosurgery, London; and Department of Neurology (N.B.), Queen's Medical Centre, Nottingham, United Kingdom.

Cathepsin A–related arteriopathy with strokes and leukoencephalopathy (CARASAL) is a recently identified cause of adult-onset cerebral leukodystrophy due to *CTSA* gene mutations described in 3 Dutch and British families.^1,2^ The clinical phenotype of CARASAL continues to be defined. Here, we report a British patient with CARASAL with brainstem dysfunction as a leading clinical issue.

## Case description.

A 48-year-old Caucasian woman (British CARASAL case^2^) presented with 5 years of deteriorating concentration and behavioral disinhibition. Recently, she had developed alternating right- or left-sided facial pain of fluctuating intensity, which was ameliorated with carbamazepine. She also reported prominent, nonpositional vertigo, difficulty following conversations in noisy environments, hyperacusis, tinnitus, and hoarseness. Her sleep was disturbed by vivid nightmares and frequent intrusive leg movements. Medical history included migraine, hypertension, sinusitis, asthma, and depression. In the family history, her father died at age 60 years after a stroke, and several paternal relatives reportedly had young onset cognitive decline, although no details were available. Her Folstein Mini-Mental State Examination score was 27/30, losing points for orientation and generation of a novel sentence, and there was bedside evidence of executive dysfunction and cognitive slowing, despite preserved memory and perceptual functions, corroborated on neuropsychometry. The general neurologic examination was unremarkable.

Brain MRI (figure, A–D) revealed diffuse, confluent T2-weighted hyperintensity of supratentorial white matter, basal ganglia, and thalamus with extensive involvement of midbrain, pons, and medulla, including auditory pathways. Pure tone audiometry revealed sensorineural hearing loss with a “cookie-bite” profile most marked for midfrequencies and transient otoacoustic emissions, consistent with mild genetic cochlear dysfunction (figure, E; table e-1 at Neurology.org/ng). Auditory evoked brainstem responses at 6 kHz showed delayed wave V (figure, F; table e-2): this was not attributable to cochlear dysfunction (given the normal wave I latency and 6–8 kHz tone detection; figure, E, tables e-1 and e-2). Furthermore, a test of spatial noise perception^3^ showed abnormal binaural interaction (table e-3), indicating (together with the brainstem evoked responses) superior olivary nuclei involvement. Peripheral vestibular assessment with electronystagmography and caloric tests revealed subtle smooth pursuit deficits, consistent with a brainstem localization. Polysomnography revealed moderate periodic limb movements of sleep and (although there was no dream enactment) loss of REM atonia, suggestive of REM sleep behavior disorder.

**Figure:**
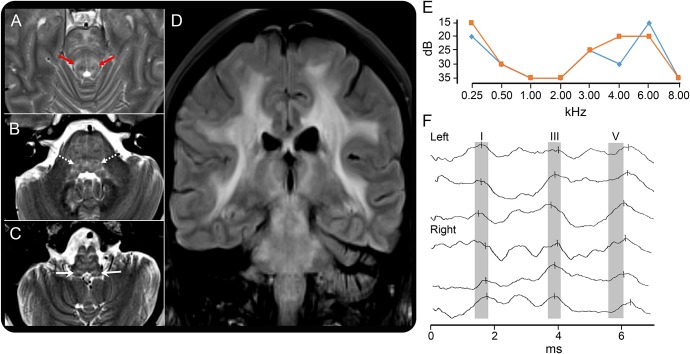
MRI and neuro-otologic findings in the present case Axial T2-weighted MRI sections through the brainstem (A–C) and a coronal fluid-attenuated inversion recovery MRI section through the thalami (D) are shown. Red arrows (A) indicate involvement of the lateral lemnisci; dotted white arrows (B) indicate involvement of the superior olivary nuclei; solid white arrows (C) indicate involvement of the dorsal, ventral, and inferior olivary nuclei. Pure tone audiometry plots (E) illustrate a “cookie-bite” profile of mild midfrequency hearing loss in both right (red) and left (blue) ears (threshold [dB] on y axis, abnormal >20 dB; table e-1). Auditory brainstem evoked responses (F); 3 recordings displayed for left (above) and right (below) ears showing that peaks (length of vertical latency marker indicates amplitude 0.2 μV) for wave V are consistently delayed beyond the normal range (gray oblongs) and normal latencies for waves I and III, indicating dysfunction of brainstem pathways between ventral cochlear nuclei and nucleus of the lateral lemniscus (table e-2). Note that these brainstem responses were evoked by a 6-kHz tone, which had a normal pure tone audiometric threshold for both ears (E).

Extensive investigations for metabolic and genetic causes of leukodystrophy proved unrevealing until the patient was ultimately shown to have a pathogenic c.973C>T, p.R325C missense mutation in the *CTSA* gene, confirming the diagnosis of CARASAL.^1^ The patient was found to share allele 123 at marker D20S838, indicating a common genetic ancestry with previously reported Dutch cases.^1^

## Discussion.

Initial descriptions of CARASAL have emphasized stroke as a dominant clinical feature and relatively indolent cognitive decline, although most patients have had memory complaints at presentation.^1^ In the series reported by Bugiani et al.,^1^ symptoms of lower cranial nerve dysfunction (including vertigo, dysphagia, dry mouth, dry eyes, central facial paresis, or dysarthria) occurred in approximately 70% of cases, and refractory hypertension was a further clinical hallmark. MRI changes commonly involve brainstem white matter with additional involvement of the thalami and other gray matter nuclei,^1^ as in our case (figure, A–D). The audiovestibular test profile here indicated a brainstem lesion, in addition to mild cochlear dysfunction of uncertain provenance. Considered collectively, the available evidence suggests a potential brainstem substrate for the symptom complex of facial pain, vertigo, hearing alterations, hoarseness, and sleep disorder exhibited by our patient and similar symptoms described in previous cases of CARASAL. The potential value of brainstem involvement in the differential diagnosis of CARASAL and related entities has not been previously emphasized.

At present, clinical differentiation of the cerebral arteriopathies exemplified by CARASAL, CARASIL, and CADASIL remains challenging.^4^ Although radiologic involvement of brainstem white matter tracts is frequently observed, neuro-otologic and other symptoms referable to brainstem structures are relatively uncommon in adult-onset leukodystrophies.^5^ In CADASIL, such symptoms are generally less salient than cognitive and psychiatric decline.^6^ Based on the clinical evidence of the present case, we propose that CARASAL should be considered in patients with adult-onset leukoencephalopathy and prominent early symptoms implicating a brainstem origin. This proposal carries the caveat that clinical experience with CARASAL remains limited (at present, a single genetic variant^1^). Although the true prevalence and mechanism remain speculative, pending further studies in larger cohorts with neuropathologic correlation, brainstem features in CARASAL could reflect dual effects of dystrophic white matter tracts and impaired perfusion of cranial nerve nuclei due to perforant arteriopathy: a mechanism previously proposed to underpin selective brainstem damage in CADASIL.^7^

## Supplementary Material

Data Supplement
