# Supplementary material for: Brainstem phenotype of cathepsin A–related arteriopathy with strokes and leukoencephalopathy
Source: Neurol Genet. 2017 Jul 6;3(4):e165. doi: 10.1212/NXG.0000000000000165 (PMC5499977; doi:10.1212/NXG.0000000000000165)
Supplement: Data Supplement [file supp_3_4_e165__index.html]

Data Supplement 

# Brainstem phenotype of cathepsin A–related arteriopathy with strokes and leukoencephalopathy

## Data Supplement

**Files in this Data Supplement:**

- e-Tables - Microsoft Word file
