# Supplementary material for: Brainstem phenotype of cathepsin A–related arteriopathy with strokes and leukoencephalopathy
Source: Neurol Genet. 2017 Jul 6;3(4):e165. doi: 10.1212/NXG.0000000000000165 (PMC5499977; doi:10.1212/NXG.0000000000000165)
Supplement: Data Supplement [file supp_3.4.e165_e-Tables.docx]

**Supplementary Data: The brainstem phenotype of CARASAL, by YT Hwang et al**

**Table e-1: Hearing assessment: pure tone audiometry**

| **Test tone frequency** (Hz) | **Left ear** (dB) | **Right ear** (dB) |
| --- | --- | --- |
| 250 | 15 | 20 |
| 500 | **30** | **30** |
| 1000 | **35** | **35** |
| 2000 | **35** | **35** |
| 3000 | **25** | **25** |
| 4000 | 20 | **30** |
| 6000 | 20 | 15 |
| 8000 | **35** | **35** |

The table shows the patient’s threshold intensity levels for detection of tones at each frequency via air conduction, for each ear. A normal tone detection threshold is 20 dB or less; values above the normal threshold are in bold. Hz = hertz; dB = decibels.

**Table e-2. Hearing assessment: auditory brainstem responses**

|  | **Left ear** (ms) | **Right ear** (ms) | **Normal range** (ms) |
| --- | --- | --- | --- |
| Ipsi I | 1.61 | 1.75 | 1.31 - 1.83 |
| Ipsi III | 3.95 | 3.97 | 3.40 - 4.08 |
| Ipsi V | **6.18** | **6.21** | 5.16 - 6.01 |
| Interval I-III | 2.31 | 2.22 | 1.83 - 2.52 |
| Interval I-V | **4.57** | 4.45 | 3.54 - 4.48 |
| Interval III-V | **2.23** | **2.23** | 1.49 - 2.19 |

The table shows the patient’s latency time for auditory brainstem waves evoked at 6 kHz. Each latency value is the average of three runs for each ear; values beyond the normal range are in bold. Ipsi = waves recorded to ipsilateral auditory stimulation; ms = milliseconds.

**Table e-3. Hearing assessment: listening in spatialized noise-sentences (LiSN-S) test**

|  | **Average score for age** | **Patient’s score (dB)** | **Variance from Average in SD** |
| --- | --- | --- | --- |
| Low Cue SRT | -0.7 | 0.2 | -0.9 |
| High Cue SRT | -14.0 | -9.4 | -2.2 |
| Talker Adv | 9.1 | 5.7 | -1.6 |
| Spatial Adv | 12.0 | 8.7 | -2.0 |
| Total Adv | 13.3 | 9.5 | -1.9 |

In this test the listener’s speech reception threshold (SRT) in decibel (dB) is established for target sentences presented in competing speech maskers (children’s stories). The target sentence is perceived as coming from directly in front of the listener while the maskers are presented from the same location as the target or from 90 degrees to the left or right of the listener, by making use of head transfer functions. The vocal identity of the speaker of the stories may be the same or different to that of the target speech. Performance on the LiSN-S is evaluated on the low-cue (same voice, same location for target and masker speech) and high-cue SRT, and on three ‘‘advantage’’ measures, i.e. the dB benefit in performance when vocal, spatial, or both types of cues are incorporated in the maskers, compared to the baseline (low-cue SRT) condition. The ability to separate the target speech from the spatially separated distracter ability is facilitated by the use of binaural cues, such as inter-aural time differences (Cameron et al., 2006), a process that is reportedly disrupted by demyelination at the level of the low brainstem at the superior olivary nuclei level (Aharonson, Furst 2001). SD = standard deviation; SRT = speech reception threshold; dB = decibels; Adv = ‘advantage’ measure

Aharonson, V., & Furst, M. (2001). A model for sound lateralization. J Acoust Soc Am., 109, 2840-51.
